# Integrated analysis of long noncoding RNA and mRNA expression profile in children with obesity by microarray analysis

**DOI:** 10.1038/s41598-018-27113-w

**Published:** 2018-06-08

**Authors:** Yuesheng Liu, Yuqiang Ji, Min Li, Min Wang, Xiaoqing Yi, Chunyan Yin, Sisi Wang, Meizhen Zhang, Zhao Zhao, Yanfeng Xiao

**Affiliations:** 1 Department of Pediatrics, The Second Affiliated Hospital of Xi’an Jiaotong University, Xi’an, Shaanxi People’s Republic of China; 2 Department of Cardiology, The First Affiliated Hospital of Xi’an Jiaotong University, Xi’an, Shaanxi People’s Republic of China

## Abstract

Long noncoding RNAs (lncRNAs) have an important role in adipose tissue function and energy metabolism homeostasis, and abnormalities may lead to obesity. To investigate whether lncRNAs are involved in childhood obesity, we investigated the differential expression profile of lncRNAs in obese children compared with non-obese children. A total number of 1268 differentially expressed lncRNAs and 1085 differentially expressed mRNAs were identified. Gene Ontology (GO) and pathway analysis revealed that these lncRNAs were involved in varied biological processes, including the inflammatory response, lipid metabolic process, osteoclast differentiation and fatty acid metabolism. In addition, the lncRNA-mRNA co-expression network and the protein-protein interaction (PPI) network were constructed to identify hub regulatory lncRNAs and genes based on the microarray expression profiles. This study for the first time identifies an expression profile of differentially expressed lncRNAs in obese children and indicated hub lncRNA RP11-20G13.3 attenuated adipogenesis of preadipocytes, which is conducive to the search for new diagnostic and therapeutic strategies of childhood obesity.

## Introduction

In the past few decades, the prevalence of overweight and obesity has also sharply increased in children and adolescents in both developed and developing countries^1^. A previous study estimated that 23.8% of boys and 22.6% of girls in developed countries and 12.9% of boys and 13.4% of girls in developing countries were overweight or obese in 2013^2^. Childhood obesity life are associated with increased risk of cardiovascular disease (CVD), diabetes mellitus, metabolic syndrome, sleep disturbances and certain cancers in adulthood^3–5^. Because of the substantial increases in prevalence and definite health risks, childhood obesity has become a serious global public health challenge. Although previous studies have indicated many factors associated with the current increase in prevalence of childhood obesity, including large birth weight, genetic influence, maternal smoking during pregnancy, lack of physical activity, nutritional factors, socio-economic position and more^6–8^, the underlying cellular and molecular mechanisms of obesity are complex and currently uncertain.

Increasing evidence suggests that besides the protein coding genes, a large number of noncoding RNAs (ncRNAs), which were formerly considered as “junk sequence”, are also involved in diverse biological processes and diseases. Long noncoding RNAs (lncRNAs) are conventionally defined as transcripts of greater than 200 nucleotides without evident protein-coding capability, as compared to other shorter ncRNAs, such as microRNA (miRNA), small interfering RNA (siRNA), and piwi-interacting RNA (piRNA)^9,10^. lncRNAs play a critical role in gene regulatory networks by a variety of mechanisms including binding with noncoding RNAs or genes, chromatin modification, splicing and translation^11,12^. Increasing evidences supported the involvement of lncRNAs in lipid metabolism including adipogenesis and metabolism^13^, adipocyte differentiation^14,15^, hepatic lipid metabolism^13,14^.

Although the role of lncRNAs in adipose metabolism has been investigated increasingly, current understanding on the function and regulatory mechanism of lncRNAs in development and progress of individual obesity is still limited. In particular, no report was made on the expression profiles of lncRNAs in childhood obesity and studying the lncRNA expression profiles in obesity children will provide some novel sights for childhood obesity. In the current study, we investigated lncRNAs and mRNAs that are differentially expressed in the obese and non-obese children by microarray. Then the mRNAs-lncRNAs regulation networks were constructed according to the microarray results and bioinformatics predictions. These findings will help us to better understand the lncRNA-related regulation mechanisms in childhood obesity.

## Materials and Methods

### Ethics Statement

The study protocol was reviewed and approved by the Ethics Committee of Xi’an Jiaotong University. Informed consent was obtained from all guardians of children participating in the study. The study was conducted in accordance with the guidelines of the Research Committee of Xi’an Jiaotong University and according to the Declaration of Helsinki. All methods were performed in accordance with the relevant protocols.

### Study Subjects and Samples

According to the BMI reference norm for Chinese children and adolescences, subjects were considered to be obese when the BMI exceeded 95th percentile of the norm^15^. Standard deviation scores (SDS-BMI) were calculated for each participant based on WHO growth reference for school-aged children and adolescents. Exclusion criteria were the presence of genetic obesity syndrome, infectious disease, autoimmune disease, tumor, and history of antibiotics, immune-modulatory drugs or Chinese traditional medicine using on recently three months, hormone deficiencies, malnutrition, type 2 diabetes (T2DM), the use of a medication that affects blood pressure, glucose or lipid metabolism. All children were pre-pubertal according to Tanner stage criteria based on testicular volume in boys and breast development in girls. The cohort of 31 obese children and 31 non-obese children were finally enrolled in study. To profile lncRNAs and mRNAs, 4 obese children and 4 non-obese children were selected randomly from the cohort for microarray. The validation of dysregulated lncRNAs for microarray and the association between the lncRNAs and obesity characteristics were then analyzed in the cohort of obese children (n = 31) and non-obese children (n = 31). Abdominal subcutaneous adipose tissue was separated bluntly during the operation, followed by washing in pre-cold PBS solution and stored in liquid nitrogen for further research. Fasting venous blood sample from each participant was collected and stored at −80 °C for biochemical measurements.

### Biochemical measurements

Fasting blood glucose (FBG), total cholesterol (TC), triglycerides (TG), high-density lipoprotein cholesterol (HDL-C) and low-density lipoprotein cholesterol (LDL-C) were measured enzymatically using an autoanalyzer (Hitachi 747; Hitachi, Tokyo, Japan). Fasting Insulin was assayed by RIA (BeiFang systems, Beijing, China), and HOMA-IR was used for estimating insulin resistance in our subjects. Plasma level of hsCRP, leptin and adiponectin was also measured by Elisa kits (Excell, Shanghai, China) with all inter-and intra assay CV < 10%.

### Total RNA Extraction

RNA was extracted from the frozen adipose tissue using Trizol reagent (Invitrogen Life Technologies, Carlsbad, CA, USA). RNA quantity and quality were measured by NanoDrop ND-1000. The high purity of the isolated RNA, as indicated by A260/280 ≥1.90, was confirmed before microarray and quantitative real-time polymerase chain reaction (qRT-PCR) experiments.

### Microarray Analysis

Microarray analysis was performed by KangChen Bio-tech (Shanghai, China). Briefly, mRNA was purified from total RNA after removal of rRNA (mRNA-ONLY™ Eukaryotic mRNA Isolation Kit, Epicentre). Then, each sample was amplified and transcribed into fluorescent cRNA along the entire length of the transcripts without 3′ bias utilizing a random priming method (Arraystar Flash RNA Labeling Kit, Arraystar). The labeled cDNA then was hybridized onto the Arraystar Human LncRNA Array v4.0. After washing the slides, the arrays were scanned on an Agilent Scanner G2505C.

Agilent Feature Extraction software (version 11.0.1.1) was used to analyze acquired array images. Quantile normalization and subsequent data processing were performed with using the GeneSpring GX v12.1 software package (Agilent Technologies). Quantile-normalized lncRNAs and mRNAs with “Present” or “Marginal” (“All Targets Value”) flags were chosen for further data analysis. Differentially expressed lncRNAs and mRNAs with statistical significance between the two groups were identified through *p*-value/FDR filtering^16^. Hierarchical clustering was performed to show the expression pattern of differentially expressed lncRNAs and mRNAs between the two groups. The microarray data have been deposited into NCBI Gene Expression Omnibus (GEO) and GEO accession is GSE104815.

### Quantitative Real-time PCR

QRT-PCR was performed using the SYBR Premix Ex TaqII (Takara, Dalian, China) according to the manufacturer’s protocol. All experiments were conducted at least three times. The expression level of each lncRNA was determined using the 2^−ΔΔCt^ method. The results were normalized to the expression of β-actin and presented as the fold change of each lncRNA. In total, the expression levels of three up-regulated and three down-regulated lncRNAs in study were measured. All primers are shown in Supplementary Table S1.

### Gene Ontology (GO) and Pathway Analysis

Differentially expressed mRNAs were selected for target prediction. GO analysis and pathway analysis were applied to determine the roles that differentially expressed mRNAs played in biological pathways or GO terms. Differentially regulated mRNAs were uploaded into the Database for Annotation, Visualization and Integrated Discovery (DAVID, https://david.ncifcrf.gov/) for annotation and functional analysis, including gene set enrichment analysis and mapping gene sets to the KEGG pathway^17,18^. Two-sided Fisher’s exact test was applied to classify the GO category with *p* < 0.05. We also employed the pathway analysis to place differentially expressed genes according to Kyoto Encyclopedia of Genes and Genomes (KEGG) (http://www.kegg.jp/kegg/) with Fisher’s exact test to identify pathways with *p* < 0.05. The enrichment score of the pathways were evaluated as previously described^19,20^.

### lncRNA-mRNA Co-expression Network

To associate the lncRNAs with direct regulated expression of target mRNAs, we constructed the lncRNA-mRNA co-expression network. The algorithm was quoted from a previously described report^21,22^. For each pair of gene analysis, the Pearson correlation was calculated and we chose those pairs with significant correlations (0.98 or greater) to construct the network. We drew the co-expression networks using Cytoscape. In this representation, each gene/lncRNA corresponded to a node and the connection between two genes was represented by an edge, indicating a strong correlation. To estimate the relative significance of a gene or lncRNA in the network, we calculated the core degree of each gene/lncRNA defined as the number of directly linked genes or lncRNAs^23^. The bigger the degree it has, the more significant it is.

### PPI Network

To investigate the proteins interaction which coded by the genes with 10 or greater core degree in lncRNA-mRNA co-expression network, the PPI network was performed automatically by STRING (Search Tool for the Retrieval of Interacting Genes/Proteins; version 10.5; https://string-db.org/)^24^. The analysis parameters were as follows: species - Homo sapiens, minimum required interaction score −0.400, active prediction methods - all. Moreover, the Molecular Complex Detection (MCODE) app was utilized to screen modules of PPI network in Cytoscape with degree cutoff = 2, node score cutoff = 0.2, k-core = 2, and max. depth = 100. The pathway analysis of genes in each module was performed by DAVID.

### Cell Culture and Oil Red O Staining

Preadipocytes SW872 were cultured in DMEM containing 10% fetal bovine serum in a humidified atmosphere (95% air and 5% CO_2_) at 37 °C and differentiated as previously described^25^. Oil red o staining for lipid accumulation were performed using described protocols^26^.

### RNAi

siRNA targeting RP11-20G13.3 was designed and synthesized by GenePharma (Shanghai, China). The sequences of the three siRNAs targeting to RP11-20G13.3 were shown in Supplementary Table S3. Based on the manufacturer’s instructions, SW872 cells were transfected with siRNA using Lipofectamine 2000 (Invitrogen, USA). Briefly, siRNA (125 pmol) and Lipofectamine 2000 (6.67 μl) in Opti-MEM I (250 ml) were dispensed onto 10^5^ cells and incubated at 37 °C in 5% CO_2_ for 4–6 h. The siRNA-Lipofectamine 2000 mixture was changed with fresh complete medium and further incubated for 24 h. Then medium was changed with oleic acid (0.6 mM, Sigma, USA) in DMEM/F12 1:1 to induce preadipocytes differentiation. The knockdown efficiency was evaluated by qRT-PCR analysis.

### Statistical Analysis

Data were analyzed using SPSS version 23.0 software. Continuous variables were presented as means ± standard deviation, means ± standard error of mean or median (interquartile range). Student’s t-test was used for comparisons between groups. Correlation analysis was utilized for expression levels of lncRNAs and clinical variables. P value < 0.05 was considered statistically significant.

## Results

### Clinical and biochemical features of included individuals

As shown as Table 1, there was no obvious difference between non-obese and non-obese groups in gender and age. Significant differences were found in BMI, SDS-BMI, waist circumstances, waist-hip ratio, SBP, fasting insulin, HOMA-IR, Triglycerides, HDL-C, LDL-C, leptin, adiponectin and leptin-to-adiponectin ratio between two groups.

**Table 1:** Clinical characteristics of study population.

	Non-obese (n = 31)	Obese (n = 31)	*p*
Sex (male/female)	21/10	21/10	1.000
Age (years)	8.14 ± 1.69	8.66 ± 2.03	0.179
BMI (kg/m^2^)	15.13 ± 1.19	22.49 ± 2.77	0.000
SDS-BMI	−0.52 (−1.41, 0.38)	2.62 (2.14, 3.09)	0.000
Waist circumference (cm)	55.48 ± 3.84	71.59 ± 11.14	0.000
Waist-hip Ratio	0.88 ± 0.06	0.92 ± 0.05	0.037
SBP (mmHg)	95 ± 7	102 ± 9	0.008
DBP (mmHg)	59 ± 6	62 ± 9	0.056
Fasting glucose (mmol/L)	4.67 ± 0.40	4.74 ± 0.40	0.082
Fasting insulin (µU/mL)	5.04 ± 3.34	12.99 ± 5.22	0.003
HOMA-IR	0.96 ± 0.41	2.63 ± 1.01	0.001
Total cholesterol (mg/dL)	3.43 ± 0.66	3.76 ± 0.51	0.107
Triglycerides (mg/dL)	0.86 ± 0.40	1.93 ± 1.11	0.000
HDL cholesterol (mg/dL)	1.37 ± 0.19	1.10 ± 0.25	0.003
LDL cholesterol (mg/dL)	1.67 ± 0.48	2.21 ± 0.57	0.008
hsCRP (ng/ml)	369.73 ± 399.65	684.52 ± 508.22	0.516
Leptin (ng/ml)	10.56 ± 8.99	28.64 ± 13.38	0.006
Adiponectin (μg/ml)	3.35 ± 1.20	2.66 ± 1.07	0.000
leptin-to-adiponectin ratio (ng/μg)	3.45 ± 2.11	14.93 ± 9.36	0.001

### Identification the different expression of lncRNAs in obese and non-obese participants

**Figure 1:**
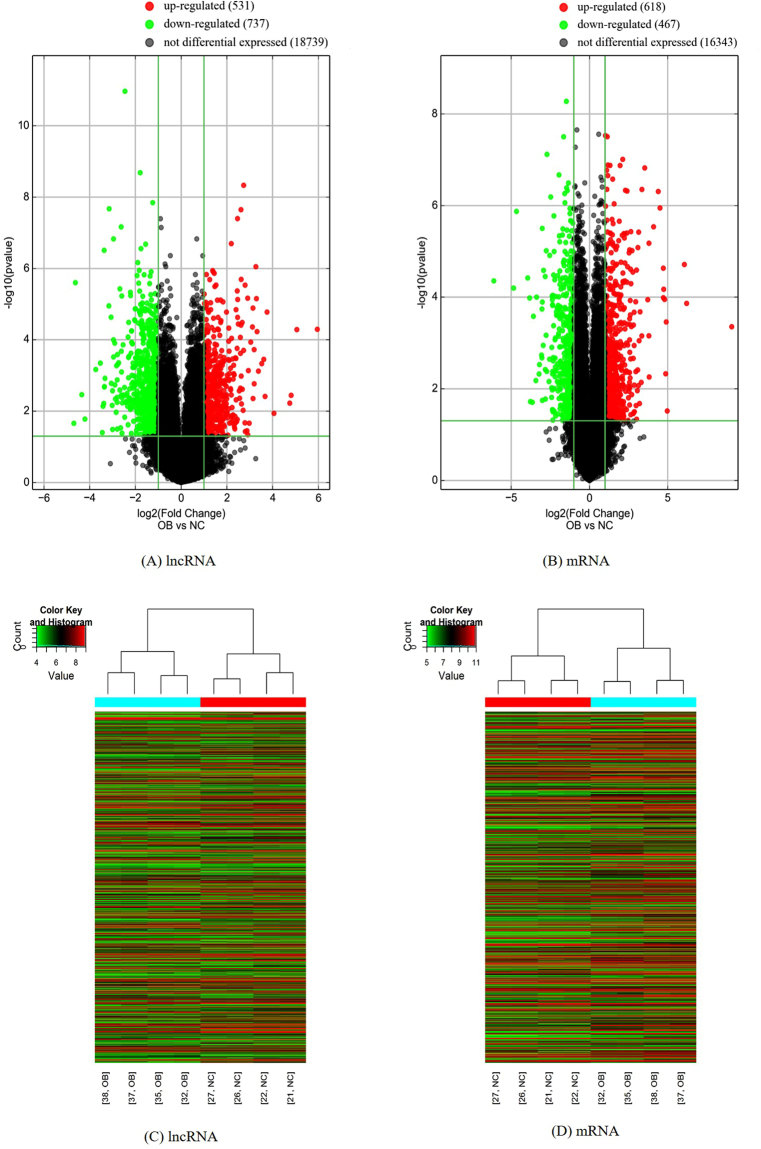
Volcano plot and Hierarchical Clustering Map. Up-regulated (red dot) or down-regulated (green dot) lncRNAs (**A**) and mRNAs (**B**) were identified between obese and non-obese group in Volcano plot. (**C**) Hierarchical Clustering analysis of differentially expressed lncRNAs between obese and non-obese group; (**D**) Hierarchical Clustering analysis of differentially expressed mRNAs between obese and non-obese group. Vertical axis represents differentially expressed genes. Red color indicates high relative expression and green color indicates low relative expression. Horizontal axis represents the names of samples. OB represent the obese individual (obese group), NC represent the non-obese individual (control group).

After the raw data were normalized (Supplementary Figure S1), the differentially expressed lncRNAs and mRNAs were identified between obese group and non-obese group. Using a 2-fold expression difference as a cutoff, a total of 1268 differentially expressed lncRNAs (531 up-regulated and 737 down-regulated) and 1085 differentially expressed mRNAs (618 up-regulated and 467 down-regulated) were discriminated between obese group and non-obese group (Fig. 1A,B). Supplementary Table S2 showed differentially expressed Top 20 lncRNAs and mRNAs identified in the microarray analysis. The Hierarchical Clustering analysis was used for analyzing the distinguishable lncRNA and mRNA expression pattern between the two groups (Fig. 1C,D).

### GO and Pathway Analysis of differentially expressed mRNAs

**Figure 2:**
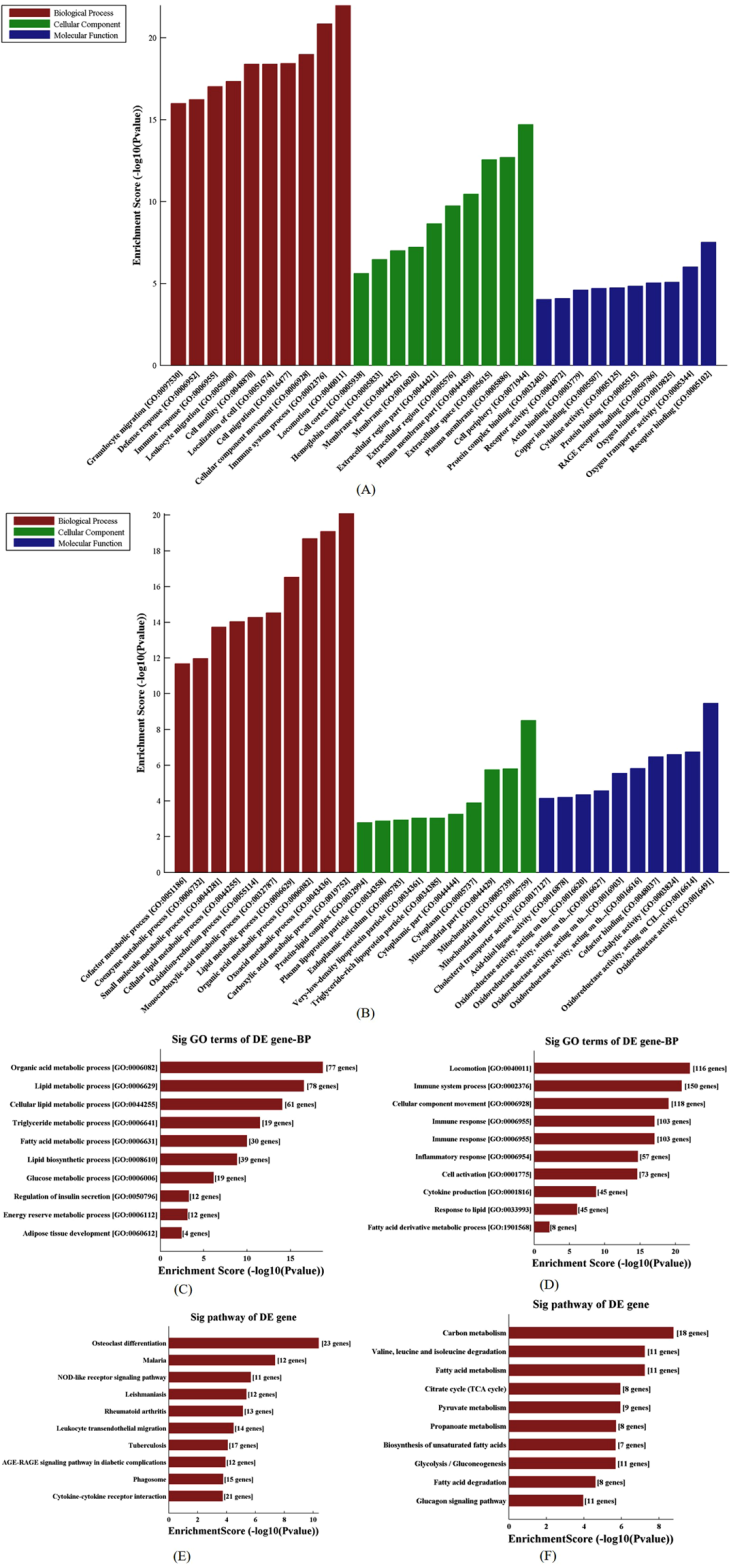
GO and pathway analysis. Red, green and blue bars represent Biological Process, Cellular Component, and Molecular Function, respectively. (**A**) Top 10 significantly up-regulated GO terms for differentially expressed mRNAs. (**B**) Top 10 down-regulated GO terms for differentially expressed mRNAs. The terms were divided into three categories, including biological process (BP, red), cellular component (CC, green), and molecular function (MF, blue). (**C**) The significantly up-regulated GO terms of BP for differentially expressed mRNAs. (**D**) The significantly down-regulated GO terms of BP for differentially expressed mRNAs. (**E**) Top 10 significantly up-regulated pathways of differentially expressed mRNAs. (**F**) Top 10 significantly down-regulated pathways of differentially expressed mRNAs.

The GO analysis is used to categorize and describe the biological functions of genes and gene products. The ontology covered three domains: Biological Process, Cellular Component and Molecular Function^27^. Top 10 enriched GO terms in three domains: Biological Process, Cellular Component and Molecular Function respectively are showed in Fig. 2A,B (separately showed in Supplementary Figure S2). According to GO analysis, up-regulated genes were mainly involved in a variety of biological processes including inflammation and lipid metabolism such as “inflammatory response (GO: 0006954)”, “fatty acid derivative metabolic process (GO: 1901568)”, and “cytokine production (GO: 0001816)” (Fig. 2C). Inversely, “lipid metabolic process (GO: 0006629)”, “glucose metabolic process (GO: 0006006)”, and “organic acid metabolic process (GO: 0006082)” were down-regulated (Fig. 2D).

Pathway analysis showed that, when comparing to controls, 41 pathways were significantly up-regulated while 45 pathways were significantly down-regulated. The most significantly up-regulated pathway was “Osteoclast differentiation (hsa04380)”, which is associated with obesity as previously reported^28^. The other up-regulated pathways included “NOD-like receptor signaling pathway (hsa04621)”, “AGE-RAGE signaling pathway in diabetic complications (hsa04933)”, and “Cytokine-cytokine receptor interaction (hsa04060)”. Meanwhile, several significantly down-regulated pathways were associated with obesity, such as “Fatty acid metabolism (hsa01212)”, “Pyruvate metabolism (hsa00620)”, and “Biosynthesis of unsaturated fatty acids (hsa01040)” (Fig. 2E,F).

### Construction of lncRNA-mRNA co-expression network and PPI network

**Figure 3:**
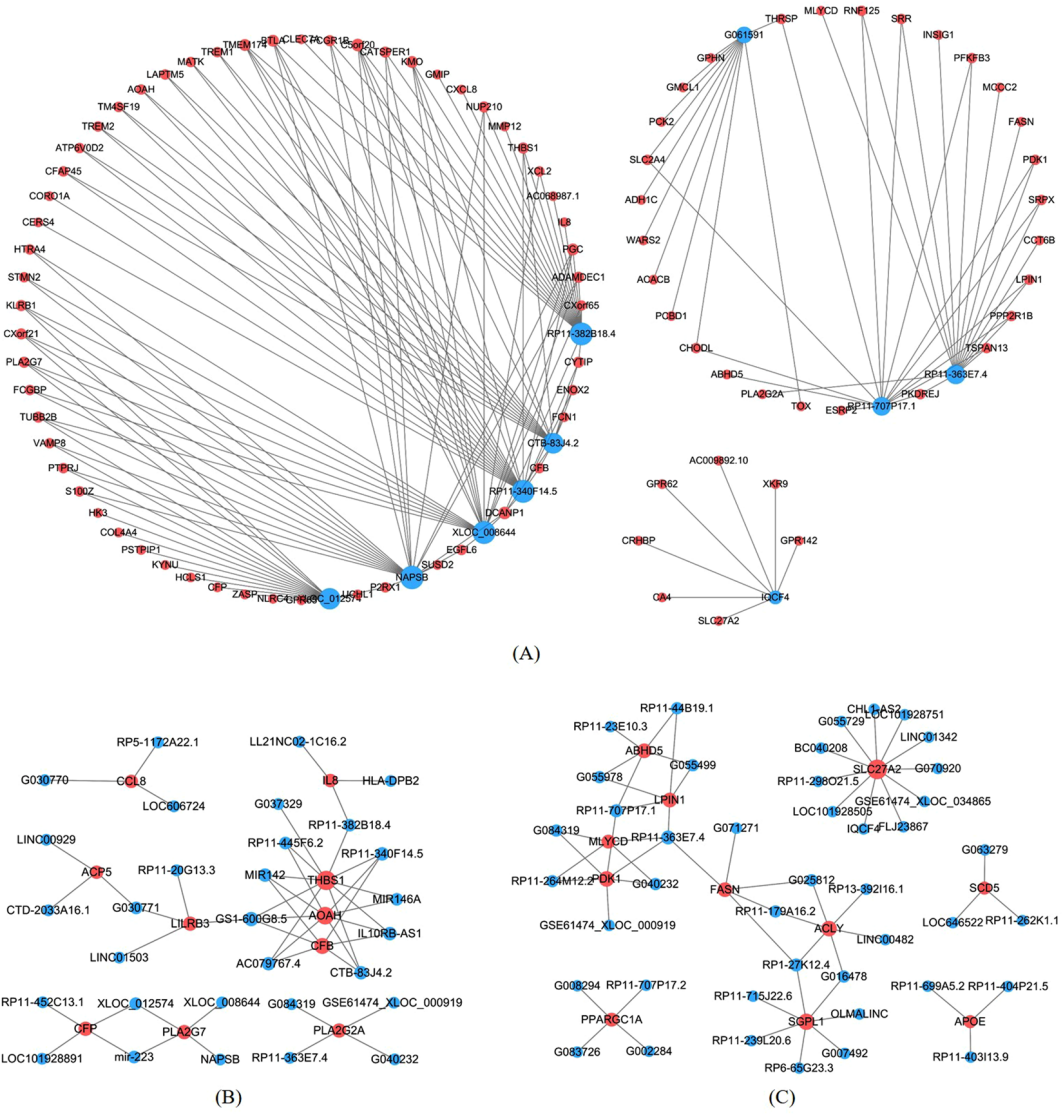
LncRNA-mRNA co-expression network. Blue and red nodes represent lncRNAs and mRNAs, respectively. Node size represents the degree. (**A**) 10 lncRNAs with higher core degrees and fold change interacted with 91 mRNAs. (**B**) 30 lncRNAs interacted with 10 mRNAs in the GO term of inflammatory response. (**C**) 43 lncRNAs interacted with 11 mRNAs in the GO term of lipid metabolic process.

The lncRNA-mRNA co-expression network was constructed to identify hub regulatory factors associated with childhood obesity. The whole profile of co-expression network was made up of 780 nodes and 2904 connections among 467 differentially expressed mRNA and 313 differentially expressed lncRNAs (See Supplementary Figure S3). The core degree was calculated to estimate the relative significance of a gene or lncRNA in the network. Ten lncRNAs with higher core degrees and fold change may be critical to the pathogenetic mechanism of childhood obesity. GO and pathway analysis of the associated genes indicated that the 10 lncRNAs were involved in multiple biological processes, such as immune response, inflammatory response, fatty acid biosynthetic process, low-density lipoprotein particle remodeling, osteoclast differentiation, AMPK signaling pathway (Table 2). Co-expression network analysis of these 10 lncRNAs and relevant 91 mRNAs were shown in Fig. 3A. Different subgroup networks according to GO term were constructed as previously reported^29^. Thirty lncRNAs interacted with 10 mRNAs in the GO term of inflammatory response (Fig. 3B), 43 lncRNAs interacted with 11 mRNAs in the GO term of lipid metabolic process (Fig. 3C).

**Table 2:** Ten lncRNAs with higher core degrees in the co-expression network.

LncRNA	Seqname	Core Degree	Fold Change	Regu-lation	GO analysis	Pathway analysis
RP11-340F14.5	ENST00000569999	28	3.50	up	immune response (GO: 0006955), inflammatory response (GO: 0006954), leukocyte migration (GO: 0050900), positive regulation of phosphatidylinositol 3-kinase signaling (GO: 0014068)	Osteoclast differentiation (hsa04380), Chemokine signaling pathway (hsa04062), NOD-like receptor signaling pathway (hsa04621)
RP11-382B18.4	ENST00000602453	27	6.56	up	immune response (GO: 0006955), inflammatory response (GO: 0006954), negative regulation of MAP kinase activity (GO: 0043407), cell differentiation (GO: 0030154)	Phagosome (hsa04145), NOD-like receptor signaling pathway (hsa04621)
NAPSB	NR_002798	25	3.02	up	immune response (GO: 0006955), inflammatory response (GO: 0006954), positive regulation of protein kinase B signaling (GO: 0051897), negative regulation of MAP kinase activity (GO: 0043407),	Phagosome (hsa04145), NOD-like receptor signaling pathway (hsa04621)
CTB-83J4.2	ENST00000596330	24	3.40	up	immune response (GO: 0006955), inflammatory response (GO: 0006954), extracellular matrix disassembly (GO: 0022617), positive regulation of phosphatidylinositol 3-kinase signaling (GO: 0014068)	Phagosome (hsa04145), NOD-like receptor signaling pathway (hsa04621)
XLOC_008644	TCONS_00018359	24	3.70	up	immune response (GO: 0006955), neutrophil chemotaxis (GO: 0030593), inflammatory response (GO: 0006954), negative regulation of MAP kinase activity (GO: 0043407),	Phagosome (hsa04145), NOD-like receptor signaling pathway (hsa04621)
RP11-559N14.5	ENST00000588565	22	3.22	down	fatty acid biosynthetic process (GO: 0006633), biotin metabolic process (GO: 0006768), fatty acid beta-oxidation using acyl-CoA dehydrogenase (GO: 0033539), regulation of glucose metabolic process (GO: 0010906)	AMPK signaling pathway (hsa04152), Adipocytokine signaling pathway (hsa04920), Insulin resistance (hsa04931)
IQCF4	NR_038214	22	2.96	down	negative regulation of actin filament polymerization (GO: 0030837), cell migration involved in sprouting angiogenesis (GO: 0002042), cellular response to calcium ion (GO: 0071277)	Null
RP11-363E7.4	ENST00000563205	20	2.05	down	low-density lipoprotein particle remodeling (GO: 0034374), fatty acid biosynthetic process (GO: 0006633), oxaloacetate metabolic process (GO: 0006107), regulation of glucose metabolic process (GO: 0010906)	AMPK signaling pathway (hsa04152), Metabolic pathways (hsa01100), Renin-angiotensin system (hsa04614)
XLOC_012574	TCONS_00026160	20	5.09	up	immune response (GO: 0006955), neutrophil chemotaxis (GO: 0030593), inflammatory response (GO: 0006954), negative regulation of MAP kinase activity (GO: 0043407),	Osteoclast differentiation (hsa04380), Lysosome (hsa04142)
RP11-707P17.1	ENST00000561007	18	3.30	down	biotin metabolic process (GO: 0006768), fatty acid biosynthetic process (GO: 0006633), regulation of glucose metabolic process (GO: 0010906), positive regulation of cellular metabolic process (GO: 0031325)	Metabolic pathways (hsa01100), Insulin signaling pathway (hsa04910), Glycolysis/Gluconeogenesis (hsa00010)

**Figure 4:**
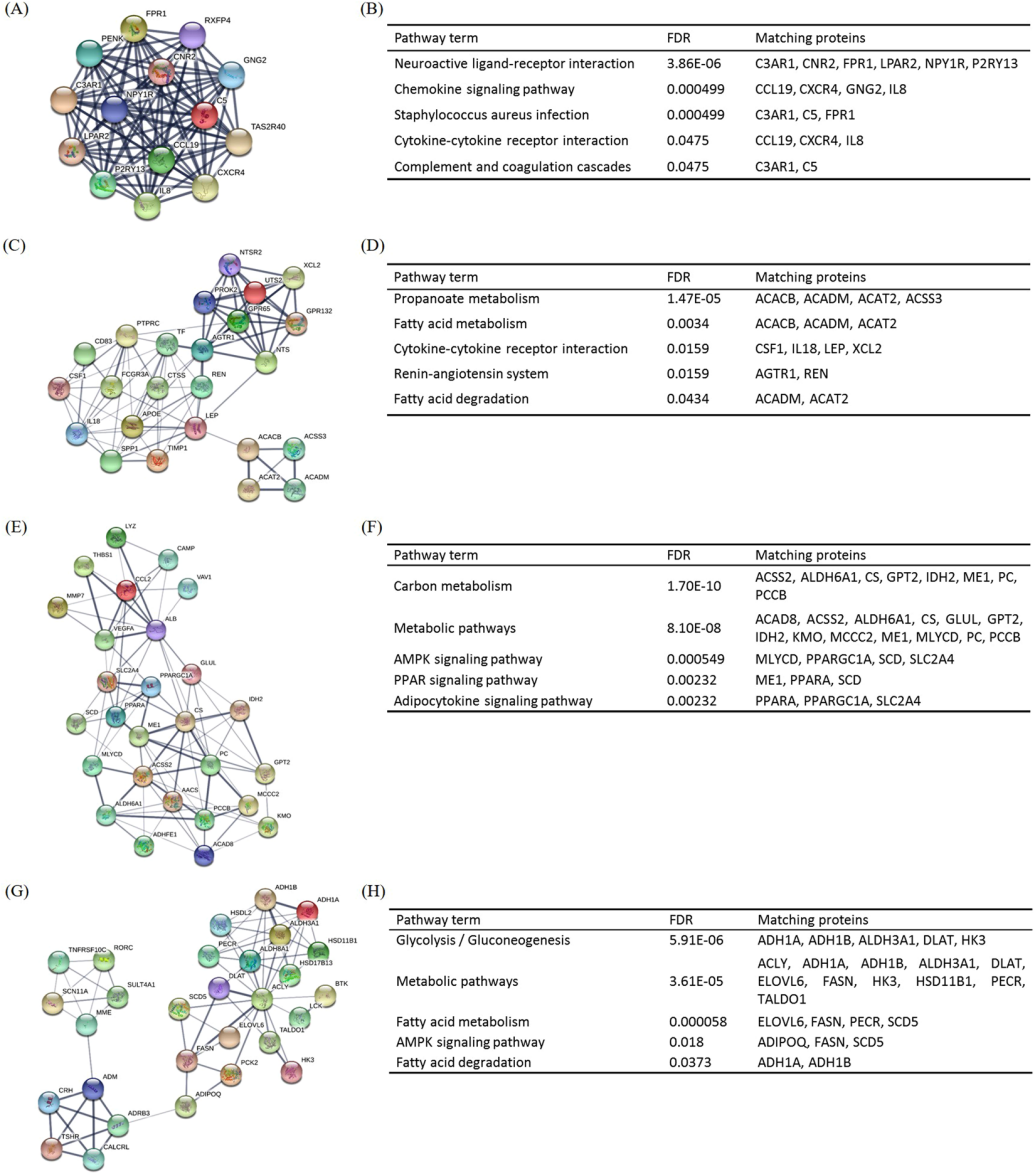
Top 4 modules from the protein-protein interaction network indicated by STRING. Line thickness indicates the strength of data support. (**A**) Module 1, (**B**) The enriched KEGG pathways of module 1. (**C**) Module 2, (**D**) The enriched KEGG pathways of module 2. (**E**) Module 3, (**F**) The enriched KEGG pathways of module 3. (**G**) Module 4, (**H**) The enriched KEGG pathways of module 4.

Protein–protein interaction networks are an important implement for the system-level understanding of cellular processes^30^. We selected genes with 10 or greater core degree in lncRNA-mRNA co-expression network, and performed the interaction network of the proteins coded by these genes via STRING. The interaction mapping showed that plenty of hub proteins^31^ might play a key role in childhood obesity, such as ACLY, ACACB, FASN, CCL19, IL8, DLAT, PC, VEGFA, ADIPOQ, and IL18 (See Supplementary Figure S4). In order to detect significant modules in this PPI network, we used MCODE plug-in. The top 4 modules were selected (Fig. 4). KEGG pathway enrichment analysis showed that these 4 modules were mainly associated with cytokine-cytokine receptor interaction, fatty acid metabolism, metabolic pathways and fatty acid degradation (Fig. 4).

### Validation of differentially expressed lncRNAs

**Figure 5:**
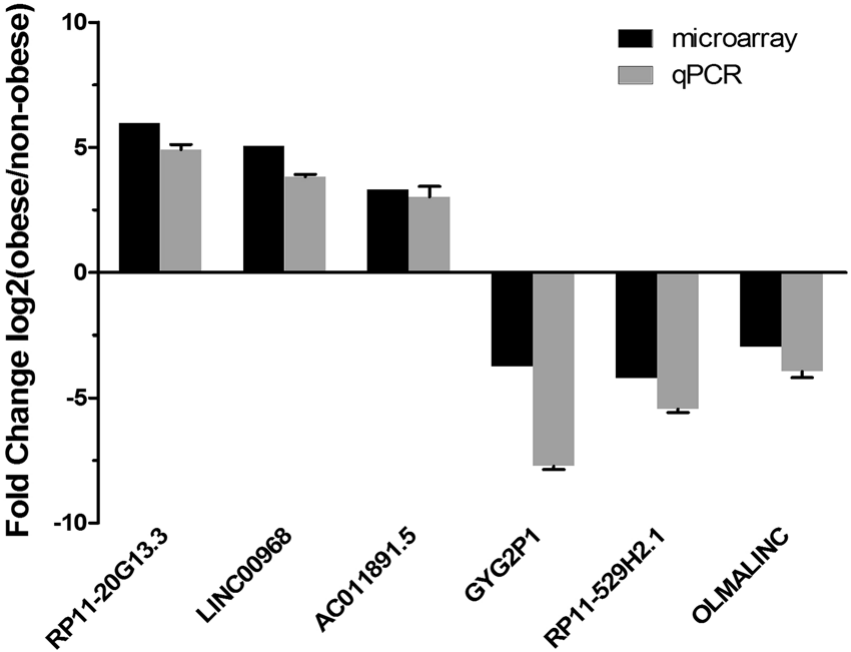
Validation of Microarray Data by qRT-PCR. Three up-regulated and three down-regulated lncRNAs were validated by qRT-PCR in adipose tissues of obese (n = 31) and non-obese (n = 31) children. The heights of the columns represent the mean expression value of log transformed fold changes (obese/non-obese).

Six lncRNAs (three up-regulated and three down-regulated) were chosen for validation of the microarray results by qRT-PCR. In qRT-PCR, the expression of lncRNA RP11-20G13.3 (ENST00000561362), LINC00968 (NR_038236) and AC011891.5 (ENST00000437088) were up-regulated, whereas GYG2P1 (NR_033667), RP11-529H2.1 (ENST00000503666) and OLMALINC (NR_026762) were down-regulated in obese group compared with non-obese group. This result was in accordance with the microarray assay (Fig. 5). The expression level of lncRNA RP11-20G13.3 was positively associated with BMI-SDS, waist circumference, waist-hip ratio, fasting insulin, LDL cholesterol, hsCRP and leptin. On the contrary, the expression level of lncRNA GYG2P1 was negatively associated with BMI-SDS, waist circumference, fasting insulin, triglycerides (Table 3).

**Table 3:** Correlation between lncRNAs relative expression level and clinical variables of obese (n = 31) and non-obese (n = 31) children. Data are showed as Mean ± SD (*p*) or R (*p*).

lncRNAs	RP11-20G13.3	GYG2P1
Relative expression level	4.90 ± 0.22(0.000)	0.13 ± 0.14(0.000)
BMI-SDS	**0.756(0.001)**	**−0.935(0.000)**
Waist circumference	**0.638(0.003)**	**−0.709(0.001)**
Waist-hip Ratio	**0.498(0.030)**	−0.342(0.165)
Fasting glucose	0.203(0.354)	−0.347(0.123)
Fasting insulin	**0.705(0.005)**	**−0.693(0.036)**
HOMA-IR	0.336(0.264)	−0.453(0.101)
Triglycerides	0.052(0.812)	**−0.472(0.031)**
Total cholesterol	0.481(0.059)	0.313(0.321)
HDL cholesterol	−0.459(0.074)	0.186(0.548)
LDL cholesterol	**0.594(0.015)**	0.313(0.297)
hsCRP	**0.688(0.033)**	−0.230(0.225)
Leptin	**0.736(0.009)**	−0.664(0.065)
Adiponectin	−0.532(0.095)	0.325(0.242)

### Knockdown RP11-20G13.3 in SW872 cells weakens adipogenesis

**Figure 6:**
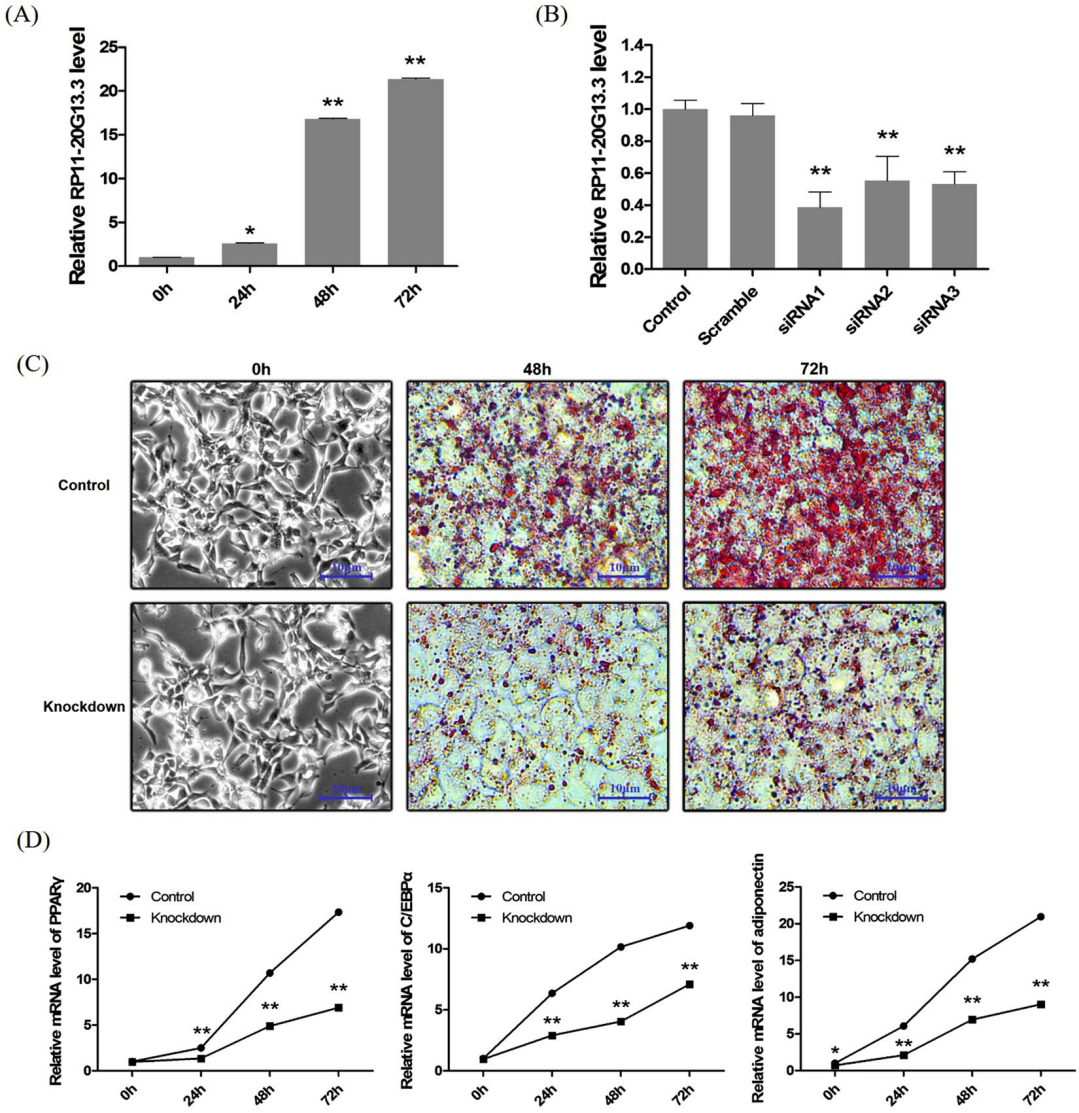
Knockdown RP11-20G13.3 in SW872 cells reduced adipogenesis. (**A**) lncRNA RP11-20G13.3 expression in the differentiation progress of SW872 preadipocytes at 0 h, 24 h, 48 h and 72 h. (**B**) Knockdown efficiencies of three kinds of siRNA targeting to RP11-20G13.3 were validated by qRT-PCR. (**C**) Oil Red O staining analysis in SW872 control and RP11-20G13.3-knockdown cells at 0 h, 48 h and 72 h. (**D**) The mRNA expression of PPARγ, C/EBPα and adiponectin during adipogenesis of control and RP11-20G13.3-knockdown cells at 0 h, 24 h, 48 h and 72 h. *P < 0.05, **P < 0.01 vs. control.

To investigate the role of hub lncRNAs in childhood obesity, we observed the functional phenotype of lncRNA RP11-20G13.3 which is the validated up-regulated lncRNAs with the highest fold change in childhood obesity. QRT-PCR analysis showed an increase in RP11-20G13.3 expression in the progress of adipocyte differentiation in SW872 cells (Fig. 6A). To further verify a role of RP11-20G13.3 in adipocyte differentiation, we conducted siRNA-mediated loss-of-function experiments. As shown in Fig. 6B, the three siRNA sequences targeting to RP11-20G13.3 evinced distinct knockdown efficiency and siRNA1-knockdown (KD) cells generated a 2.59-fold decrease in RP11-20G13.3 expression over controls. We then chose siRNA1 for next experiments. We observed that knockdown of RP11-20G13.3 significantly prevented adipocyte differentiation, as evidenced by oil red O staining (Fig. 6C). Moreover, we examined the mRNA expression of PPARγ, C/EBPα and adiponectin, markers of adipocyte differentiation, by qRT-PCR at 0 h, 24 h, 48 h and 72 h. QRT-PCR analysis showed that knockdown RP11-20G13.3 reduced the mRNA levels of PPARγ, C/EBPα and adiponectin during adipocyte differentiation (Fig. 6D).

## Discussion

Increasing evidence has identified more and more lncRNA species and biology, however there is limited knowledge of lncRNA regulation function in human adipose tissue or their role in human disease^32^. In the present study, we successfully explored the lncRNA expression profile in adipose tissue of obese and non-obese using microarray technology. This is the first time to report lncRNA expression and lncRNA-mRNA co-expression in the obesity children. Based on the microarray data, we identified 1268 lncRNAs and 1085 mRNAs which were differentially expressed between the two groups (fold change ≥2.0, p < 0.05), suggesting a large amount of lncRNAs may be linked to childhood obesity.

In order to study the biological functions of these lncRNAs, we utilized GO analysis and pathway analyses. According to the GO analysis, the main biological processes involving the differentially expressed lncRNAs included “inflammatory response”, “cytokine production”, “lipid metabolic process”, “glucose metabolic process” and “organic acid metabolic process”. Inflammatory response linked to obesity has been well studied that increase of the pro-inflammatory cytokines while decrease of anti-inflammatory cytokines causes chronic low-grade inflammation in adipose tissue^33,34^. Moreover, previous study has indicated the abnormal organic acid metabolism in obese children^35^. Pathway analysis showed that the genes associated with the lncRNAs differentially expressed mainly involved “Osteoclast differentiation”, “AGE-RAGE signaling pathway in diabetic complications”, “Fatty acid metabolism”, “Pyruvate metabolism” and “Biosynthesis of unsaturated fatty acids”.

Consistent with our findings, a study in obese mice reported that “osteoclast differentiation” pathway was highly enriched in high-fat diet obese mice^36^. Evidences have showed that obesity is associated with lower bone formation and inferior bone quality^28^, and clarify biological functions of lncRNAs associated with osteoclast differentiation contribute to treatment of these disorders. In addition, the decrease in unsaturated fatty acids is correlated with insulin resistance in childhood obesity, which shows the unsaturated fatty acids playing a significant role in childhood obesity^37^. Surprisingly, we have also found several enriched pathway of which the roles have not been studied in the childhood obesity development until now, such as carbon metabolism and malaria, which needs further study. Besides, we selected five important GOs/pathways and linked them to some lncRNAs through lncRNA-mRNA network, and speculated the potential functions of these lncRNAs for the further studies.

To our knowledge, lncRNAs play a biological function via a variety of mechanisms including binding with noncoding RNAs or genes, chromatin modification, splicing and translation instead of a common way like genes or miRNAs^11,38^. Besides, a growing number of evidence shows that lncRNA may play a critical regulatory function in adipose tissue by an interacting network^39^. In the present study, we constructed lncRNA-mRNA co-expression network to identify hub lncRNAs associated with childhood obesity. The results showed that the differentially expressed lncRNAs interacted with the differentially expressed genes, which indicates the complexity of molecular mechanisms for childhood obesity. Interestingly, 10 lncRNAs with higher core degrees and fold change were involved in immune response, inflammatory response, lipid metabolism, osteoclast differentiation, glycometabolism and other biological processes, which may be critical to the mechanism of childhood obesity.

The PPI network indicated that plenty of hub proteins might play a key role in childhood obesity, such as ACLY, ACACB, FASN, CCL19, IL8, DLAT, PC, VEGFA, ADIPOQ, and IL18. Interestingly, previous study revealed a crosstalk between acetylation and ubiquitylation of ACLY in the regulation of fatty acid synthesis and cell growth^40^. However, verification of its function associated with childhood obesity needs further experiments. Adipose tissue is also an abundant source of inflammatory cytokines and associated with a chronic low-grade inflammatory state^33^. Evidence supports our results that IL8, IL18 and adiponectin are differentially expressed in subcutaneous adipose tissue compared with visceral adipose tissue in in severe obese individuals^41^. Moreover, IL-18 Production is regulated by the NLRP1 Inflammasome in the context of metabolic stress, Preventing Obesity and Metabolic Syndrome^42^. The predicted interaction networks indicated a number of proteins involved in multiple biological processes such as cytokine-cytokine receptor interaction, fatty acid metabolism, metabolic pathways and fatty acid degradation, which suggested new directions for future experimental research.

To validate the dependability of the microarray, we chose three up-regulated and three down-regulated lncRNAs for validation by qRT-PCR. We indicated that lncRNA RP11-20G13.3, LINC00968 and AC011891.5 were up-regulated, whereas GYG2P1, RP11-529H2.1 and OLMALINC were down-regulated in obese group compared with non-obese group. The result was in accordance with the microarray. Furthermore, we observed the correlation between expressions of lncRNA RP11-20G13.3, GYG2P1 and Clinical features, respectively. The results showed that lncRNA RP11-20G13.3 was positively associated with BMI-SDS, waist circumference, waist-hip ratio, fasting insulin, LDL cholesterol, hsCRP and leptin. On the contrary, the expression level of lncRNA GYG2P1 was negatively associated with BMI-SDS, waist circumference, fasting insulin, triglycerides. This result reveals that these two lncRNAs may play a critical role in pathogenetic mechanism of childhood obesity. Future studies may confirm the molecular regulation mechanism of these two candidate lncRNAs.

By the qRT-PCR analysis, we evidenced that the expression of lncRNA RP11-20G13.3 increased during SW872 differentiation. In order to explore whether RP11-20G13.3 regulates adipogenesis, we knocked down the expression of RP11-20G13.3 in SW872 cells using siRNA-liposome. Results showed that RP11-20G13.3 knockdown significantly reduced lipid accumulation and decreased mRNA expression of PPARγ, C/EBPα and adiponectin, which are markers of adipogenic differentiation, during adipocytes differentiation. Several lncRNAs have been reported to play important roles in adipogenesis, such as NEAT1, PU.1 antisense lncRNA, and lncRNA U90926^43–45^. However, these studies have not observed hub lncRNAs in the regulation of adipogenesis in Human adipocytes. In this study, we indicated that lncRNA RP11-20G13.3, hub lncRNA in the obesity children, attenuated adipogenesis of preadipocytes.

We recognize that our research has several limitations. In the present study, the sample size was still small and a larger sample cohort is needed for verification. Although we identified the potential lncRNAs and associated pathways associating with childhood obesity, the molecular mechanisms of these potential regulators in childhood obesity are still required to be investigated *in vivo* or *vitro*.

## Conclusion

This study for the first time provides an expression profile of differentially expressed lncRNAs and mRNAs in obese children. Bioinformatic analysis results have identified a number of potential regulators and pathways, providing a novel perspective on the mechanisms of childhood obesity, which is conducive to the search for new diagnostic and therapeutic strategies. LncRNA RP11-20G13.3, hub lncRNA in the childhood obesity recognized by microarray analysis, attenuated adipogenesis of preadipocytes. Because the bioinformatic analysis is a preliminary tool for mechanisms, further study is required to investigate the molecular regulation mechanisms of these potential regulators and pathways involved in childhood obesity development and progress.

## Electronic supplementary material

Supplementary Info
