# Supplementary material for: Integrated analysis of long noncoding RNA and mRNA expression profile in children with obesity by microarray analysis
Source: Sci Rep. 2018 Jun 8;8:8750. doi: 10.1038/s41598-018-27113-w (PMC5993825; doi:10.1038/s41598-018-27113-w)
Supplement: Supplementary file 1 — Supplementary Info [file 41598_2018_27113_MOESM1_ESM.pdf]

**Title Page**

**Title:** Integrated analysis of long noncoding RNA and mRNA expression profile in children with obesity by microarray analysis

**Authors:** Yuesheng Liu[1], Yuqiang Ji[2], Min Li[1], Min Wang[1], Xiaoqing Yi[1], Chunyan Yin[1], Sisi Wang[1], Meizhen Zhang[1], Zhao Zhao[2] and Yanfeng Xiao[1].

1 Department of Pediatrics, The Second Affiliated Hospital of Xi'an Jiaotong University, Xi'an, Shaanxi, People's Republic of China.

2 Department of Cardiology, The First Affiliated Hospital of Xi'an Jiaotong University, Xi'an, Shaanxi, People's Republic of China.

**Correspondence:** Professor Yanfeng Xiao, Department of Pediatrics, The Second Affiliated Hospital of Xi'an Jiaotong University, 157 Xiwu Road, Xi'an, Shaanxi, 710004, China. E-mail: xiaoyanfeng0639@sina.com

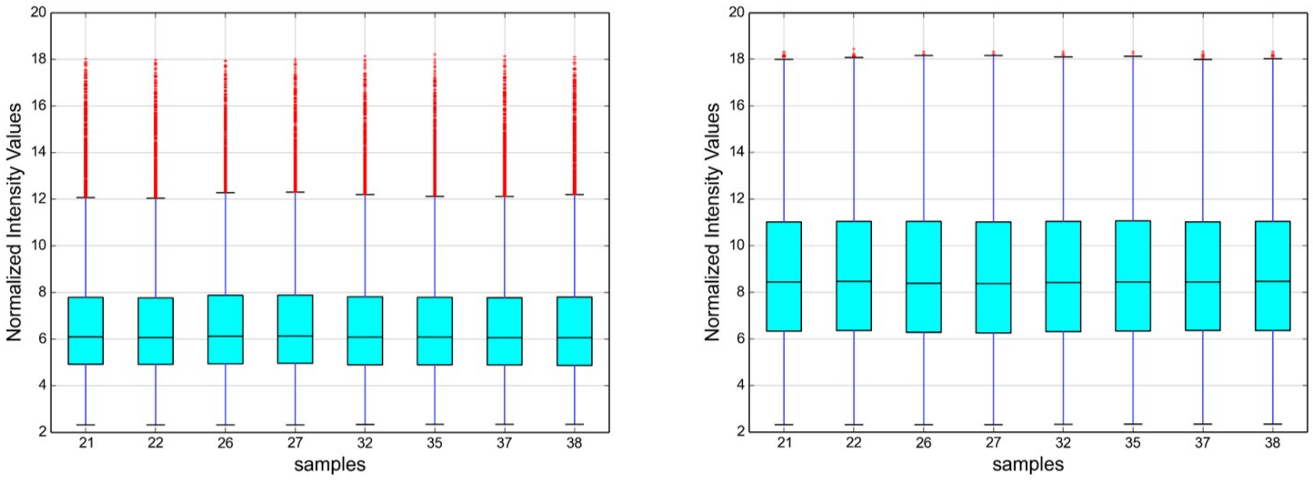


(A) lncRNA (B) mRNA

Supplementary Figure S1. Box plot. Box plot is used to observe and compare the distributions of lncRNA (A) and mRNA (B) expression values of the samples after normalization.

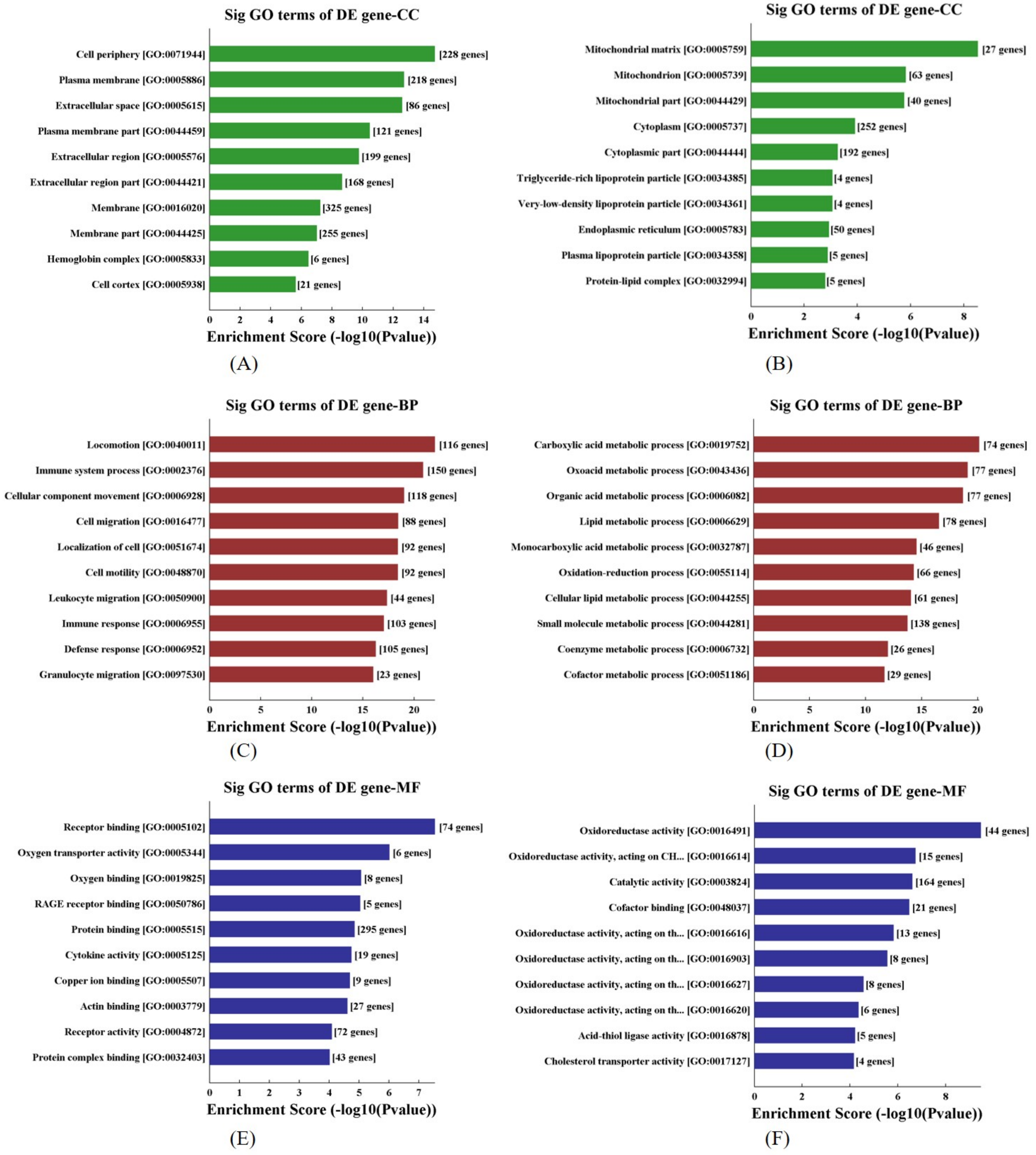


Supplementary Figure S2. GO analysis. The GO terms were divided into three categories, including cellular component (CC, green), biological process (BP, red), and molecular function (MF, blue). (A) (C) (E) Top 10 significantly up-regulated GO terms for differentially expressed mRNAs. (B) (D) (E) Top 10 down-regulated GO terms for differentially expressed mRNAs.

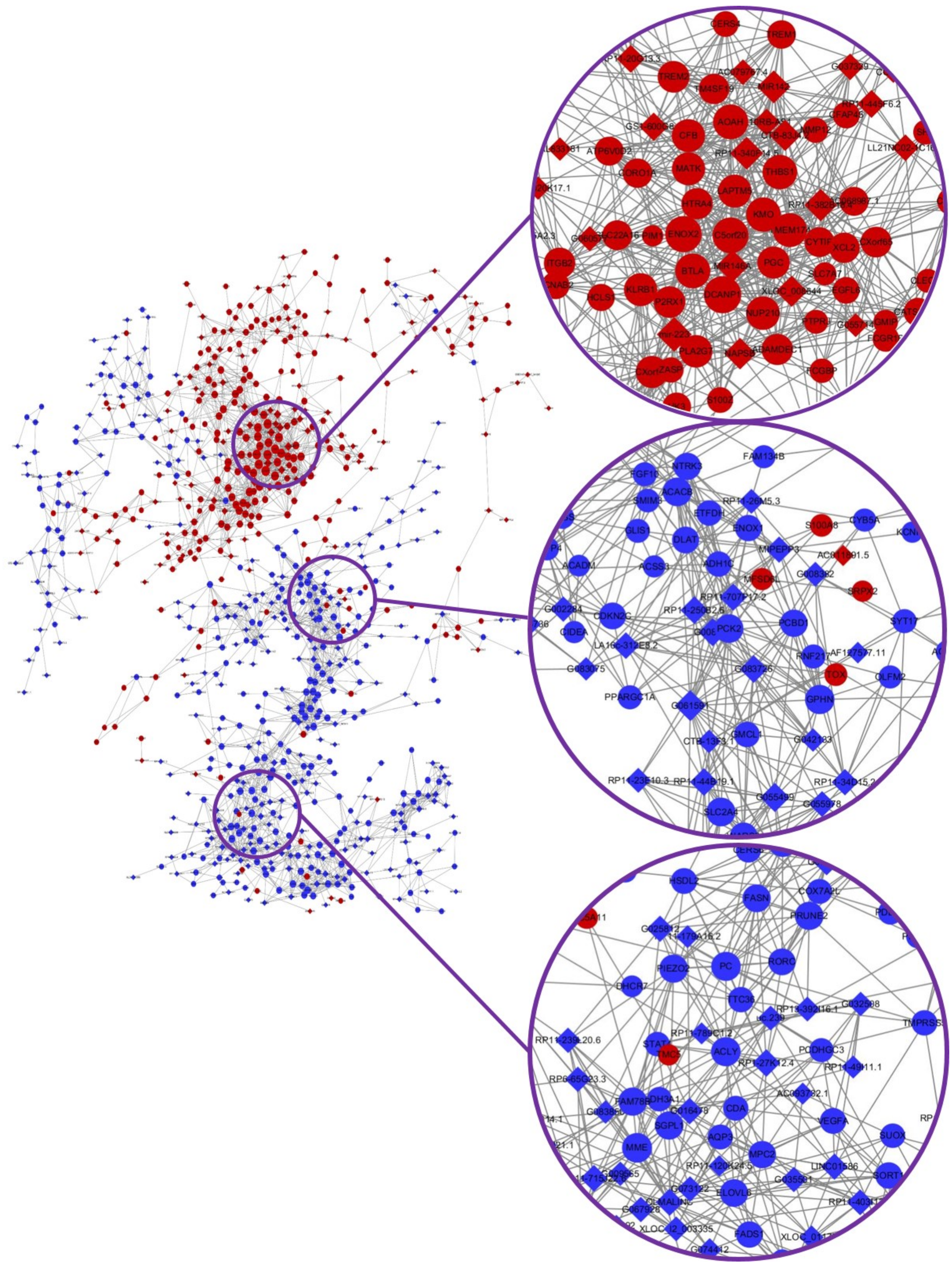


Supplementary Figure S3. LncRNA-mRNA Co-expression Network Analysis. Diamond and round nodes represent lncRNAs and mRNAs, respectively. Red color represents up-regulated lncRNAs or mRNA, blue color represents down-regulated lncRNAs or genes, node size represents the degree.

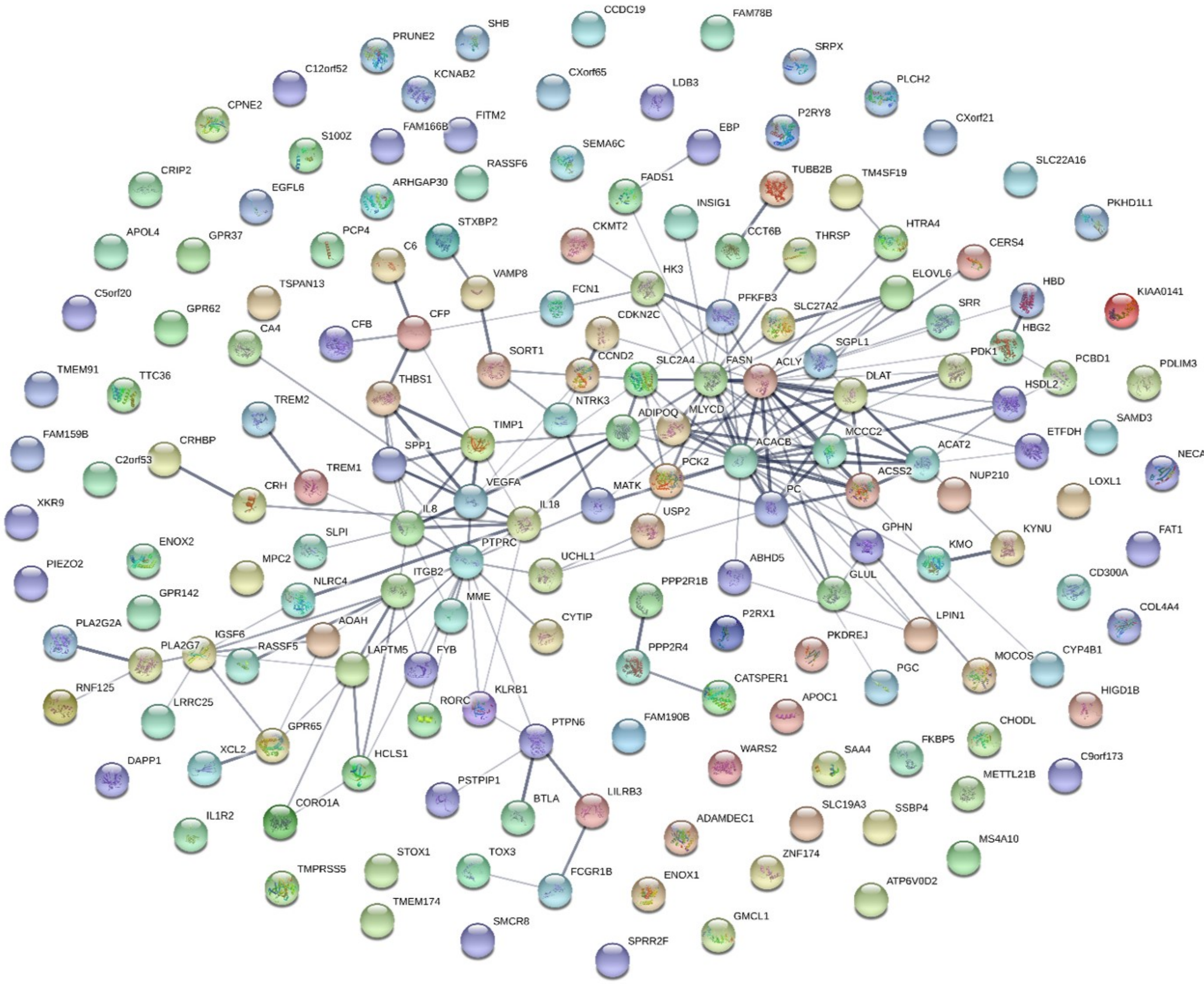


Supplementary Figure S4. The protein-protein interaction network was indicated by STRING. Line thickness indicates the strength of data support.

Supplementary Table S1 PCR primers used for validation studies (F forward primer and R reverse primer)

| Gene Symbol | Seqname | Primer Sequence (5′–3′) |
|---|---|---|
| Primers for lncRNAs | | |
| RP11-20G13.3 | ENST00000561362 | F: TCTGGAAGGAGTGTCGGTCT<br>R: CGTGTTCACAGATTGGGAGA |
| LINC00968 | NR_038236 | F: ACCATCCCATTGAGAACCAA<br>R: CGAAAGGCTGGAAGTGTCAT |
| AC011891.5 | ENST00000437088 | F: TGACCCAATTCTGACATTTGC<br>R: CCATCCTTTGGGCAGGTTA |
| GYG2P1 | NR_033667 | F: TCAGCCTCCCAAGTAGCTGT<br>R: CAGCCTGTGTCTCCTCAGTG |
| RP11-529H2.1 | ENST00000503666 | F: AGGAGAATGGTGAAGGCAGA<br>R: TGCCGAAGCAGTTTAATCCT |
| OLMALINC | NR_026762 | F: AGACCCAGGACAGGAGGACT<br>R: ATTGGCAAGATGTTCCTTGG |
| Primers for internal control | | |
| β-actin | NM_001101 | F: TGGCACCCAGCACAATGAA<br>R: CTAAGTCATAGTCCGCCTAGAAGCA |

Supplementary Table S2 Top 20 differentially expressed lncRNAs and mRNAs identified in the microarray analysis

| Seqname | GeneSymbol | Regulation | Fold Change | P-value | FDR |
|---|---|---|---|---|---|
| **lncRNA** | | | | | |
| ENST00000561362 | RP11-20G13.3 | up | 62.073459 | 5.091E-05 | 0.0044477 |
| NR_038236 | LINC00968 | up | 33.386909 | 5.195E-05 | 0.0044994 |
| NR_003276 | CES1P1 | up | 16.690729 | 0.0115549 | 0.0873364 |
| NR_073133 | CFAP221 | up | 13.518736 | 1.677E-05 | 0.0026989 |
| NR_120685 | LINC01503 | up | 10.410163 | 0.0007574 | 0.0189901 |
| ENST00000437088 | AC011891.5 | up | 9.9810222 | 5.911E-05 | 0.0047879 |
| ENST00000412485 | GS1-600G8.5 | up | 9.154965 | 0.0017162 | 0.0300396 |
| NR_103548 | LUCAT1 | up | 9.1455208 | 0.0001887 | 0.0091612 |
| ENST00000440518 | RP11-445F6.2 | up | 8.840088 | 0.0044422 | 0.0508146 |
| ENST00000455011 | RP3-460G2.2 | up | 8.8201881 | 1.294E-05 | 0.0025023 |
| ENST00000429420 | RP11-34D15.2 | down | 24.651778 | 2.508E-06 | 0.0013561 |
| NR_047508 | LINC00417 | down | 20.328518 | 0.003467 | 0.0447801 |
| ENST00000503666 | RP11-529H2.1 | down | 18.46743 | 0.0167242 | 0.108032 |
| NR_033667 | GYG2P1 | down | 13.30221 | 0.0006811 | 0.017929 |
| NR_038455 | LOC100507389 | down | 10.421125 | 0.0060959 | 0.0615999 |
| ENST00000431500 | AC012456.4 | down | 10.045824 | 0.001206 | 0.0247734 |
| NR_109780 | LOC100506860 | down | 8.935325 | 1.124E-05 | 0.002406 |
| NR_103750 | KIF25-AS1 | down | 8.8505841 | 2.126E-08 | 7.523E-05 |
| ENST00000454777 | RP11-310H4.1 | down | 8.3031096 | 0.001671 | 0.0296382 |
| NR_034024 | LINC00347 | down | 8.0836874 | 0.0069124 | 0.0665209 |
| **mRNA** | | | | | |
| NM_015507 | EGFL6 | up | 545.82779 | 0.000442 | 0.0080862 |
| NM_018965 | TREM2 | up | 74.072179 | 0.0001368 | 0.0043592 |
| NM_001040058 | SPP1 | up | 67.028393 | 1.937E-05 | 0.0015585 |
| NM_007029 | STMN2 | up | 31.351211 | 0.0304885 | 0.1079769 |
| NM_138461 | TM4SF19 | up | 29.984489 | 0.0003478 | 0.007191 |
| NM_014479 | ADAMDEC1 | up | 29.267564 | 0.0047086 | 0.0335633 |
| NM_006786 | UTS2 | up | 27.906376 | 0.000112 | 0.0038121 |
| NM_001135690 | PENK | up | 26.828298 | 6.733E-05 | 0.0029088 |
| ENST00000326958 | AC026703.1 | up | 26.413032 | 0.000103 | 0.0036529 |
| NM_022351 | NECAB1 | up | 26.354291 | 2.332E-05 | 0.0017329 |
| NM_001164442 | FAM159B | down | 69.141144 | 4.413E-05 | 0.0023419 |
| NM_024090 | ELOVL6 | down | 28.804723 | 6.334E-05 | 0.0028489 |
| NM_024944 | CHODL | down | 25.331879 | 1.344E-06 | 0.0004463 |
| NM_173605 | KCNRG | down | 15.399287 | 3.811E-05 | 0.0021914 |
| NM_001080430 | TOX3 | down | 14.156503 | 0.0001043 | 0.0036782 |
| NM_003645 | SLC27A2 | down | 13.693693 | 0.0190868 | 0.0807388 |
| NM_000065 | C6 | down | 12.520815 | 0.0197195 | 0.0824871 |
| NM_001164310 | FAM166B | down | 11.977411 | 0.0002622 | 0.0061741 |
| NM_001657 | AREG | down | 10.72578 | 0.0066176 | 0.0413842 |
| NM_002588 | PCDHGC3 | down | 10.398921 | 0.0001 | 0.0036038 |

Supplementary Table S3 The sequences of the three siRNAs targeting to RP11-20G13.3

| siRNA | Sequence (5′–3′) |
|---|---|
| siRNA1 | sense: GCUGUUAGUACUGCUUUCATT |
| | antisense: UGAAAGCAGUACUAACAGCTT |
| siRNA2 | sense: GACAGCAGUUCCCUGGCUUTT |
| | antisense: AAGCCAGGGAACUGCUGUCTT |
| siRNA3 | sense: CCCAAUCUGUGAACACGAATT |
| | antisense: UUCGUGUUCACAGAUUGGGTT |
